# Navigating the Gene Co-Expression Network and Drug Repurposing Opportunities for Brain Disorders Associated with Neurocognitive Impairment

**DOI:** 10.3390/brainsci13111564

**Published:** 2023-11-07

**Authors:** Mathew Timothy Artuz Manuel, Lemmuel L. Tayo

**Affiliations:** 1 School of Chemical, Biological, and Materials Engineering and Sciences, Mapúa University, Manila City 1002, Philippines; mtamanuel@mymail.mapua.edu.ph; 2 School of Graduate Studies, Mapúa University, Manila City 1002, Philippines; 3 Department of Biology, School of Medicine and Health Sciences, Mapúa University, Makati City 1200, Philippines

**Keywords:** hub genes, Alzheimer’s disease, drug repurposing, neurocognitive disorder, microarray, WGCNA

## Abstract

Neurocognitive impairment refers to a spectrum of disorders characterized by a decline in cognitive functions such as memory, attention, and problem-solving, which are often linked to structural or functional abnormalities in the brain. While its exact etiology remains elusive, genetic factors play a pivotal role in disease onset and progression. This study aimed to identify highly correlated gene clusters (modules) and key hub genes shared across neurocognition-impairing diseases, including Alzheimer’s disease (AD), Parkinson’s disease with dementia (PDD), HIV-associated neurocognitive disorders (HAND), and glioma. Herein, the microarray datasets AD (GSE5281), HAND (GSE35864), glioma (GSE15824), and PD (GSE7621) were used to perform Weighted Gene Co-expression Network Analysis (WGCNA) to identify highly preserved modules across the studied brain diseases. Through gene set enrichment analysis, the shared modules were found to point towards processes including neuronal transcriptional dysregulation, neuroinflammation, protein aggregation, and mitochondrial dysfunction, hallmarks of many neurocognitive disorders. These modules were used in constructing protein-protein interaction networks to identify hub genes shared across the diseases of interest. These hub genes were found to play pivotal roles in processes including protein homeostasis, cell cycle regulation, energy metabolism, and signaling, all associated with brain and CNS diseases, and were explored for their drug repurposing experiments. Drug repurposing based on gene signatures highlighted drugs including Dorzolamide and Oxybuprocaine, which were found to modulate the expression of the hub genes in play and may have therapeutic implications in neurocognitive disorders. While both drugs have traditionally been used for other medical purposes, our study underscores the potential of a combined WGCNA and drug repurposing strategy for searching for new avenues in the simultaneous treatment of different diseases that have similarities in gene co-expression networks.

## 1. Introduction

Brain disorders encompass a wide array of neurological and psychiatric conditions, manifesting as neurodegenerative diseases, psychiatric disorders, or even as malignancies like brain tumors [1,2]. While the underlying causes and clinical presentations may vary widely, a recurring theme in many of these disorders is the onset of neurocognitive impairment. Neurocognitive disorders encompass a range of conditions that adversely lead to the impairment of cognitive abilities, including memory, executive function, and attention, often due to underlying neurological disorders, damage, or even dementia, where the decline in cognitive function is severe enough to interfere with daily life [1,2]. Among many others, these disorders include Alzheimer’s disease (AD), Parkinson’s disease with Dementia (PDD), HIV-associated neurocognitive disorders (HAND), and glioma, each of which presents with distinct causes and clinical manifestations [3,4]. Moreover, the molecular changes resulting from these diseases occur in various brain regions, involving various genes and proteins.

For example, AD is a leading cause of dementia, characterized by a progressive decline in cognitive functions, including memory, language skills, and the ability to perform routine tasks. Concurrently, AD is also a neurodegenerative disease, marked by the accumulation of amyloid-beta plaques and tau tangles, leading to neuronal loss and brain atrophy, and later on, worsening dementia. These pathological hallmarks contribute to the cognitive decline observed in AD and are often used as targets for therapeutic interventions. The dual characterization of AD as both a neurodegenerative disease and a form of dementia underscores the complexity of its pathophysiology and the need for multi-faceted approaches in research. Genetic factors such as mutations in the *APP*, *PSEN1*, and *PSEN2* genes have been implicated in the rapid accumulation of beta-amyloid protein [5]. PD is initially a movement disorder, with symptoms including tremors, rigidity, and bradykinesia, and primarily involves the loss of dopaminergic neurons in the substantia nigra [6,7]. Alpha-synuclein aggregation is a hallmark, and several genes have been reported to be involved in its pathogenesis, such as *SNCA*, *LRRK2*, *VPS35*, *PARK2*, *PINK1*, and *DJ-1* [8]. Parkinson’s Disease Dementia (PDD) occurs in the later stages of PD and involves not just motor but also cognitive dysfunction severe enough to interfere with daily life [6]. The cholinergic system is often compromised, adding another layer of complexity to its molecular underpinning [9]. HAND represents a spectrum of neurocognitive impairments associated with HIV infection, ranging from asymptomatic neurocognitive impairment to HIV-associated dementia [10]. It is caused by the direct effects of HIV infection in the brain or the result of infection from opportunistic organisms, which leads to synaptic damage and neuronal loss. Different from other forms of dementia, it involves viral proteins like Tat and gp120 affecting neuronal function [11]. Glioma, despite not being categorized under neurodegenerative diseases like AD or PDD, is a type of brain tumor that causes a variety of neurological symptoms [12]. In the case of glioma, the infiltrating nature of the tumor along with edema may contribute to the cognitive impairment. Gliomas are known for their highly infiltrative nature, invading surrounding healthy brain tissue and often creating a pro-inflammatory microenvironment by secreting cytokines like IL-6 and TNF-alpha [12]. These cytokines can activate microglia, leading to a chronic state of inflammation that is also a hallmark of neurodegenerative diseases. The activated microglia can release more pro-inflammatory cytokines and reactive oxygen species, exacerbating both tumor growth and neuronal damage [13].

Despite these diseases having fundamentally different etiologies and pathomechanisms, both past and emerging evidence suggests that these diseases may share common molecular mechanisms [10]. For example, neuroinflammation, oxidative stress, and mitochondrial dysfunction have been implicated in the pathogenesis of all four diseases [12,14,15,16]. Additionally, protein misfolding and aggregation, a hallmark of AD and PDD, have also been observed in HAND and glioma [7,15,17,18,19]. For example, misfolded and aggregated proteins often share common pathways for clearance, such as autophagy and the ubiquitin-proteasome system, suggesting a potential point of therapeutic intervention for multiple neurocognitive disorders [20]. Neuroinflammation is mediated by activated microglia and astrocytes. In both Alzheimer’s and Parkinson’s diseases, as well as in HAND, neuroinflammatory processes brought about by microbial infections contribute to neuronal damage contribute to neuronal damage [2,21]. Genes like APOE and HLA-DR in both AD and PDD have been implicated in modulating immune responses in the brain, providing another layer of genetic commonality [13].

Oxidative stress is another shared mechanism, often resulting from mitochondrial dysfunction. Genes like *PINK1* and *PARK2* in PDD and *APP* in AD have roles in mitochondrial function. The imbalance in reactive oxygen species (ROS) production and clearance is a shared feature across these diseases, affecting similar cellular pathways, including the MAPK and NF-κB pathways.

An advanced systems biology technique called weighted gene co-expression network analysis (WGCNA) is used to collectively describe the correlation patterns of genes across different samples and provide information on the overall gene expression landscape in specific conditions. Using WGCNA, researchers can find potential biomarkers or therapeutic targets linked to specific biological processes or diseases by classifying genes into clusters, called modules, based on their expression levels [22]. Due to its capacity to shed light on the complicated interactions between genes, especially in the setting of complex disorders, WGCNA has recently experienced tremendous growth in popularity. Its use goes beyond conventional analyses of differential expression, providing a more comprehensive perspective of gene interactions and their collective impact on diseases [23]. The need for robust statistical workflows like WGCNA capable of analyzing enormous and complex genomic data are growing as DNA microarray and high-throughput sequencing technologies become more widely available [24]. One interesting feature of WGCNA is module preservation analysis, a preservation statistic used to quantitatively measure the preservation of modules from a reference dataset to another dataset [24]. Drug repurposing, the process of identifying new therapeutic uses for approved drugs, has emerged as a promising approach in the field of drug discovery due to its potential to speed up drug development and reduce costs [25]. A pivotal tool in this field of study is the Connectivity Map (CMap), which provides an extensive database of gene expression profiles in cells treated with many different small molecules, and the Molecular Signatures Database (MSigDB), which enables the discovery of possible therapeutic candidates based on gene signatures [26]. 

WGCNAs ability to cluster genes into modules based on their strong co-expression patterns provides a nuanced understanding of the molecular underpinnings of diseases. By identifying hub genes in these modules that are perturbed in each disease state, one can gain insight into the key pathways and processes disrupted in that condition [27]. Once these disease-associated gene modules are identified through WGCNA, they can be pipelined to drug-repurposing methods, allowing the screening of compounds based on molecular and gene signatures that modulate gene expression changes of the disease-associated patterns [27].

Herein, microarray datasets corresponding to AD (GSE4281), PD (GSE7621), HAND (GSE35864), and glioma (GSE15824) acquired from the Gene Expression Omnibus (GEO) database, a repository for high-throughput gene expression and genomics studies supported by the National Center for Biotechnology Information (NCBI) [28], were used to perform a cross-study WGCNA to identify clusters (modules) of highly correlated genes that have high preservation across the gene expression datasets. This study highlights four gene co-expression modules that were highly preserved across all datasets. Modules were found to have common and distinct features based on characterization via functional annotation clustering and the construction of protein-protein interaction (PPI) networks. Over-represented biological processes, cellular components, molecular functions, and the Kyoto Encyclopedia for Genes and Genomes (KEGG) pathways database all point toward critical terms being investigated for their significant implications in neurocognitive disorders like mitochondrial dysfunction, transcriptional deregulation, chromatin structure formation, protein phosphorylation, and growth signaling pathways. Identifying key gene networks associated with these mechanisms may help further evolve our understanding of their involvement in various brain diseases. Moreover, the identified hub genes within each module were used as gene signatures for drug repurposing studies on the Drug Repurposing Encyclopedia (DRE) webserver. Drug repurposing through comparison of gene signatures with the DRE database was used to generate a set of drugs that are highly associated with the hub genes. Candidate drugs that modulate the expression of the hub genes were identified. The approach and findings could provide interesting insights into the mechanisms of neurocognitive impairment across different disorders and open new avenues for how new drugs will be developed.

## 2. Materials and Methods

### 2.1. Dataset Gathering and Pre-Processing

The following microarray data were acquired from the National Center for Biotechnology Information-Gene Expression Omnibus (NCBI GEO) online database (https://www.ncbi.nlm.nih.gov/geo/ (accessed on 23 April 2023)) for WGCNA analysis: HIV-associated neurodegenerative disease (GSE35864) [29], Alzheimer’s disease (GSE5281) [30], Parkinson’s disease (GSE7621) [31], and glioma (GSE15824) [32] (Table 1). Datasets were selected carefully based on tissue source, microarray platform used (GPL570—HG-U133 Plus 2 Affymetrix Human Genome U133 Plus 2.0 Array), and number of samples (*n* > 15). The HAND dataset contains 72 samples composed of post-mortem brain tissue from normal, HIV-infected, HIV-dementia, and HIV-w/substantial neurocognitive impairment and encephalitis subjects. The PD dataset contains 25 samples from post-mortem brain tissue from normal and Parkinson’s disease subjects. The AD dataset contains 161 samples from normal and Alzheimer’s disease patients. The GM dataset contains 35 samples from frozen brain tissue from control patients and those with primary glioblastoma, secondary glioblastoma, astrocytoma, and oligodendroglioma.

Raw data were normalized using the Robust Multi-array Average (RMA) method from the “affy” package in Bioconductor using R version 4.3.1 for Windows (http://www.bioconductor.org (accessed 23 April 2023)). The expression data were filtered to retain only the genes that had a mean and variance higher than the 20% percentile cut-off across all samples in each dataset. Additionally, only probes present across all datasets were used, excluding control probes. Log-2 transformation of each dataset was applied, and genes and samples with significant numbers of missing values were filtered out using the “goodSamplesGenesMs” function of the WGCNA R package. Genes that remained present across all datasets were used for the remainder of the experiments. Furthermore, preliminary sample clustering based on Euclidean distance was performed to construct a sample dendrogram for each dataset to exclude noticeable outliers.

### 2.2. Weighted Gene Co-Expression Network Analysis (WGCNA)

#### 2.2.1. Approximating the Scale-Free Network

The appropriate soft-thresholding power, β, was determined by plotting the scale-free topology fit against power indices of 1–20 using the “pickSoftThreshold” function of the WGCNA R package and choosing the lowest power where the scale-free topology criterion is met. Finally, the chosen β was evaluated by plotting the approximate straight-line relationship using the values for soft connectivity, *k*, for each dataset. To briefly assess the comparability of datasets prior to network construction, the ranked expression plots and ranked soft connectivity plots between each dataset were constructed.

#### 2.2.2. Network Construction and Module Identification

The chosen soft-thresholding power was used to calculate the adjacency matrices of each dataset through Pearson’s correlation with the network type set to “signed”. Afterward, the adjacency matrices were used to calculate the topological overlap measure (TOM) dissimilarities for performing hierarchical clustering of the genes. The tips of the branches within the constructed gene dendrograms correspond to highly correlated genes that can be clustered into modules.

The “hybrid tree cut” function within the dynamic tree-cutting algorithms was used to cluster and identify the modules [33]. The “deep split” parameter was varied from 0 to 3 to control the sensitivity of the branch splitting.

#### 2.2.3. Module Preservation and Module Membership

Weighted gene co-expression network preservation of PD in GM, HAND, and AD at the module level was qualitatively and quantitatively measured using the “modulepreservation” function within the WGCNA R package. The network type was set to “signed”, with the number of permutations set to 100 and the minimum module size set to 30 genes. Modules found to be highly preserved across all datasets were chosen as modules of interest.

Genes within each module of interest were identified by calculating their eigengene-based connectivity, or kME, using the “moduleEigengenes” function from the WGCNA R package. The correlations between each gene’s expression profile and the module eigengene were then used as a measure of module membership. Genes were considered hubs if they had high kME ranks in multiple networks and were extracted for functional enrichment. 

### 2.3. Functional Annotation and Pathway Enrichment of Highly Preserved Modules

The Database for Annotation, Visualization, and Integrated Discovery (DAVID) webserver (https://david.ncifcrf.gov (accessed 12 May 2023)), containing a set of annotation tools for deciphering and correlating functions of genes based on existing literature, was used to perform functional annotation clustering [34]. The Gene Ontology (GO) database for biological processes (BP), molecular functions (MF), and cellular components (CC) were selected as categories for functional annotation clustering. The GO database is a repository for the fundamental properties and functions of genes and the proteins they encode [35]. The classification stringency was set to “medium”, and only statistically significant GO terms (*P adj. <* 0.05) with clustering enrichment scores above 1.3 were considered for analysis.

Pathway enrichment using the Kyoto Encyclopedia of Genes and Genomes (KEGG) database was used to further deduce details about the biological, genomic, chemical, and systemic functions of the modules of interest [36]. Top-scoring KEGG terms and those that clustered with significant GO terms were prioritized.

### 2.4. Protein-Protein Interaction (PPI) Networks and PPI-Based Hub Genes

Genes within each highly preserved module were accounted for their corresponding protein-protein interactions using the Search Tool for the Retrieval of Interacting Genes/Proteins (STRING) database to create PPI networks [37]. PPI networks for each module of interest were constructed with a minimum interaction score of 0.7 (high confidence). The constructed networks were imported to Cytoscape for the identification of hub genes using the Cytohubba feature in terms of degree, maximum neighborhood component (MNC), and edge percolated component (EPC) [38].

### 2.5. Signature-Based Approach for Drug Repurposing

Screening for repurposeable drugs was performed using the recently launched Drug Repurposing Encyclopedia (DRE) (https://www.drugrep.org (accessed 18 May 2023)), an interactive web server that makes use of over 198 million significant drug-signature associations from the Molecular Signatures Database (MSigDB) and over 30,000 drug-associated transcription profiles from Connectivity Map (CMap) [39]. The top ten (10) hub genes in each module were used as gene signatures and grouped into upregulated and downregulated based on differential expression analysis (DEA) using GEO2R (https://www.ncbi.nlm.nih.gov/geo/geo2r (accessed 18 May 2023)) before being submitted to the DRE webserver for drug repurposing analysis. Drugs that were either in experimental stages or withdrawn were excluded from the results. Only results with known mechanisms and those with Benjamini and Hochberg’s false discovery rates (FDR) less than 0.05 were considered. In each group, the top five (5) highest-ranking drugs based on Tau scores were recorded.

## 3. Results

### 3.1. Weighted Gene Co-Expression Network Analysis (WGCNA)

#### 3.1.1. Data Pre-Processing

**Figure A2:**
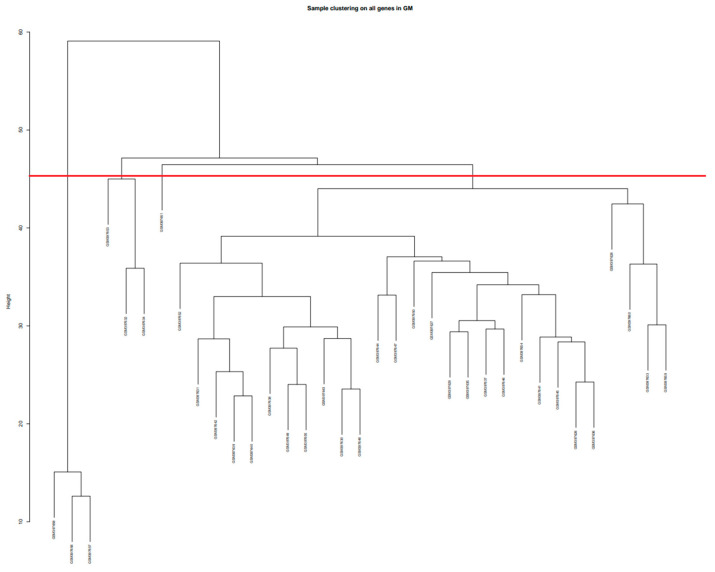
Preliminary sample clustering dendrogram of the glioma dataset based on Euclidean distance to remove sample outliers. The smaller clusters cut by the red line were treated as sample outliers.

**Figure A3:**
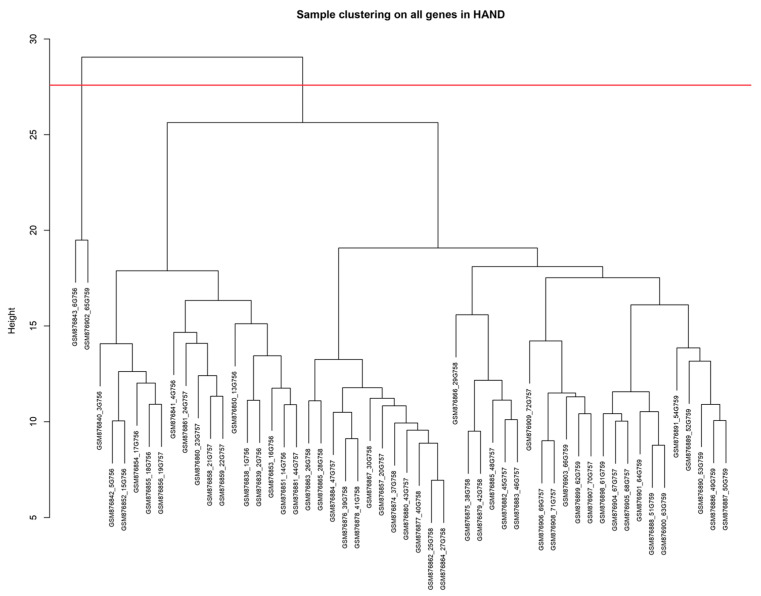
Preliminary sample clustering dendrogram of the HIV-associated Neurocognitive Disorder (HAND) dataset based on Euclidean distance to remove sample outliers. The smaller clusters cut by the red line were treated as sample outliers.

**Figure A4:**
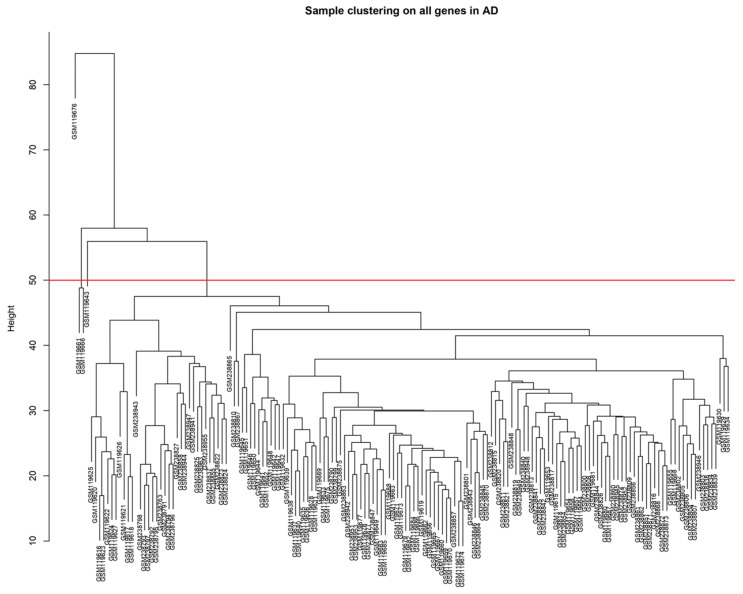
Preliminary sample clustering dendrogram of the Alzheimer’s Disease dataset based on Euclidean distance to remove sample outliers. The smaller clusters cut by the red line were treated as sample outliers.

**Figure A5:**
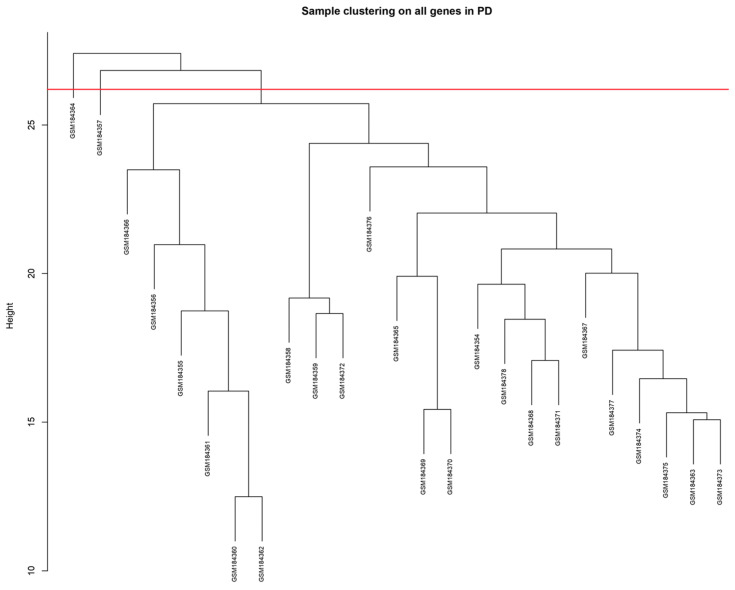
Preliminary sample clustering dendrogram of the Parkinson’s dataset based on Euclidean distance to remove sample outliers. The smaller clusters cut by the red line were treated as sample outliers.

**Figure A6:**
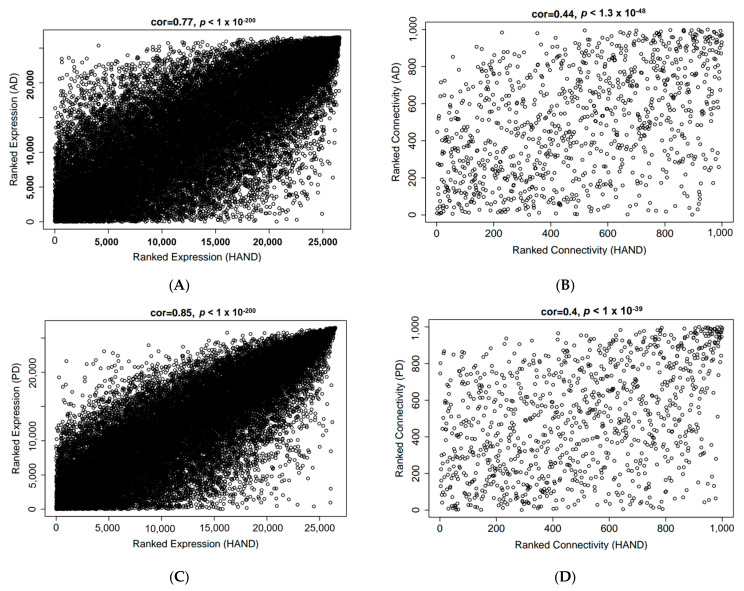

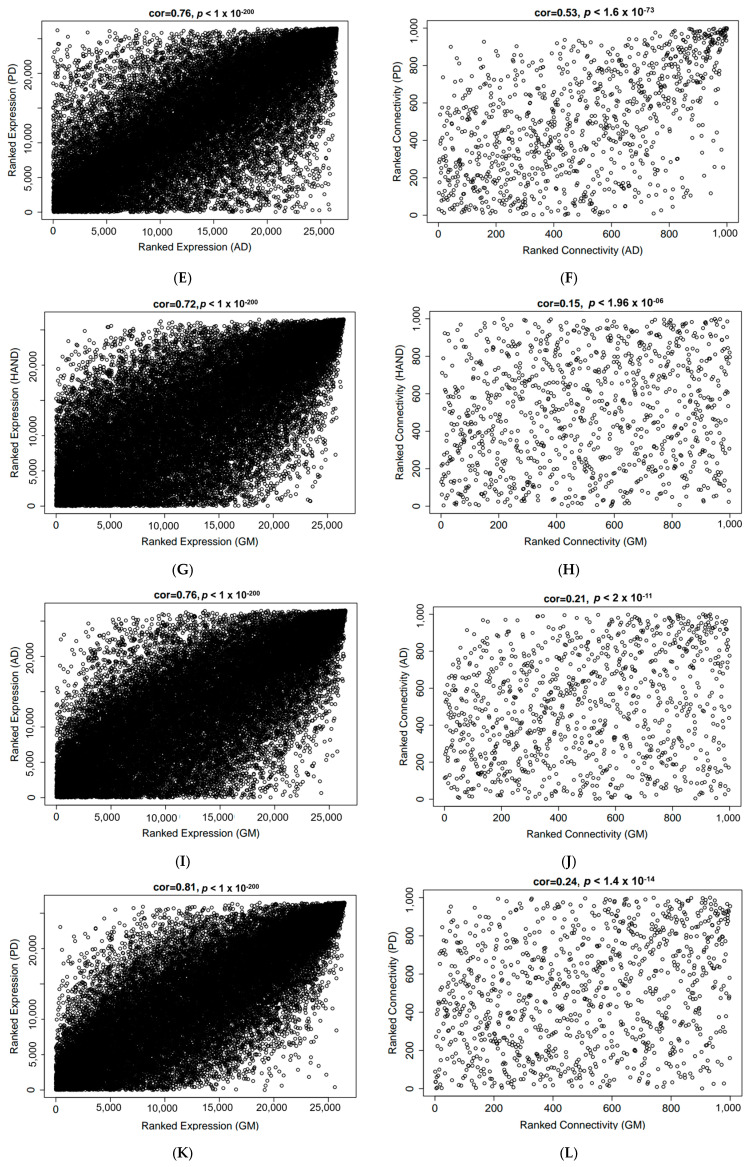
Summary of ranked expression plots and ranked connectivity plots, respectively, for each dataset comparison for (**A**,**B**) AD vs. HAND, (**C**,**D**) PD vs. HAND, (**E**,**F**) PD vs. AD, (**G**,**H**) HAND vs. Glioma, (**I**,**J**) AD vs. Glioma, and (**K**,**L**) PD vs. Glioma.

A total of 26,545 genes remained after data filtering. Preliminary clustering of samples was used to remove outliers in each dataset. The red line indicates the cut line for removing outliers. The smaller clusters cut by these lines were treated as outliers. Samples that were observed to fall under well-defined clusters were retained for further analyses (Figure A2, Figure A3, Figure A4 and Figure A5). The remaining genes and samples were used to construct the weighted gene co-expression networks. The results for checking the comparability between each dataset are displayed in Figure A6. A positive correlation was observed for all comparisons, suggesting the comparability of datasets. The plot and statistics were better for ranked expression than ranked connectivity, a pattern that is recurrent in WGCNA studies [7].

#### 3.1.2. Approximating Scale-Free Networks

**Figure 1:**
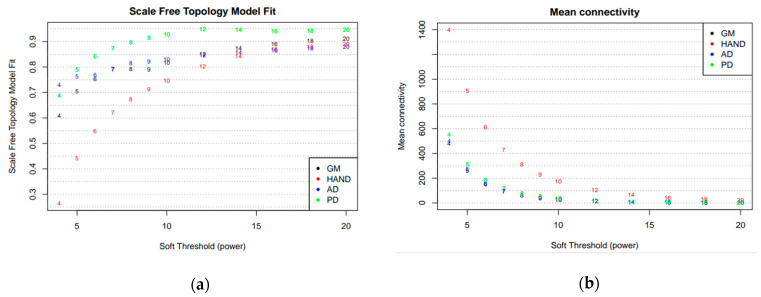
A summary of the network indices to approximate scale-free topology (**a**) and a plot of the mean connectivity (**b**) are measures of the average number of connections per gene in the networks and indicate the overall interconnectedness.

For each dataset, the scale-free topology fit index was plotted for values of β from 1 to 20 and selected as the power at which the curve flattens out and reaches close to 1 (Figure 1a). This measures how well the network conforms to a scale-free topology, a property of many biological networks [40]. Higher fit indices lead to better fits toward a scale-free topology at the expense of over-penalizing relatively weaker correlations, leading to an inaccurate network that may not reflect underlying biology [41]. The scale-free topology fit index of AD and GB had a value of 0.7 even at low power values. The almost flat curve implies that their scale-free topology does not change significantly with increasing power, suggesting a robust scale-free structure not sensitive to the choice of β. For PD, a high index was achieved at a power of 10, where the curve begins to significantly flatten out. However, HAND attained low scale-free topology fit indices even at relatively high β. The chosen β must balance emphasizing strong correlations while preserving scale-free topology to detect meaningful modules. Hence, scale-free topologies for all datasets were approximated at β = 10.

The mean connectivity of the networks is also shown in Figure 1b. At the chosen soft-thresholding power of 10, the networks for all datasets still exhibited relatively high mean connectivity, indicating the networks were well-connected and not overly fragmented, suggesting that the resulting networks balance the need to emphasize strong correlations while maintaining levels of interconnectedness [41].

**Figure 2:**
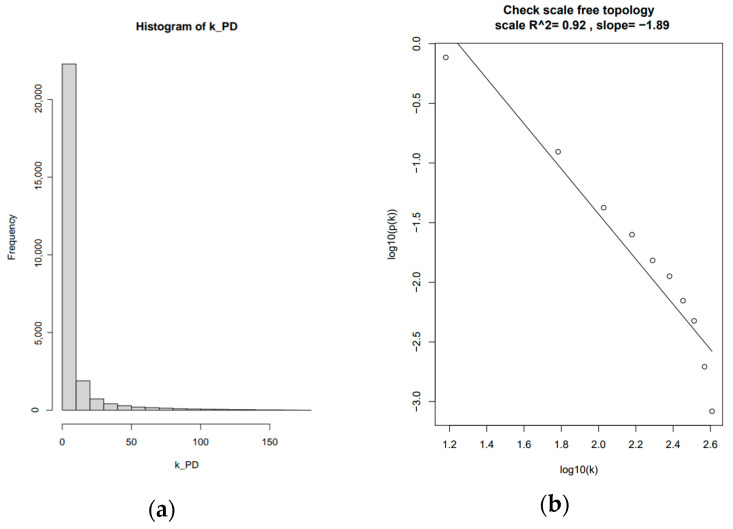
(**a**) histogram of network connectivity, k, is used to calculate (**b**) the approximate straight-line relationship for the PD dataset with beta = 10 as the soft-thresholding power.

To further validate the choice of β, the histogram of connectivity, k, was examined (Figure 2a), and its values were used to plot the approximate straight-line relationship of a dataset using 10 as the soft-thresholding power (Figure 2b). The scale-free network was best represented using the PD dataset, having the highest R^2^ value of 0.92.

#### 3.1.3. Network Construction and Module Identification

In WGCNA, a meta-analysis approach can be conducted in multiple ways [27,42,43,44]. One is by setting one dataset as a reference and projecting the module eigenvalues of the other datasets against the reference [17,27]. The criteria for choosing the reference affect the robustness of the network and can be based on many factors, such as the number of samples within a dataset, how well the scale-free network was approximated, and how defined the clustering is in the TOM-based gene dendrograms [41]. PD is one of the most prevalent neurodegenerative disorders, second only to AD. In North America, approximately 1 million people were living with Parkinson’s in 2018 [45]. Parkinson’s well-studied involvement of key pathways such as mitochondrial dysfunction, alpha-synuclein aggregation, and neuroinflammation, which are also implicated in other neurodegenerative diseases, makes it a suitable reference for a cross-disease WGCNA approach [46,47]. Additionally, the network constructed using the PD dataset was the most satisfactory in terms of scale-free topology model fit and TOM-based gene clustering and was therefore used as a reference for module preservation analysis. 

**Figure 3:**
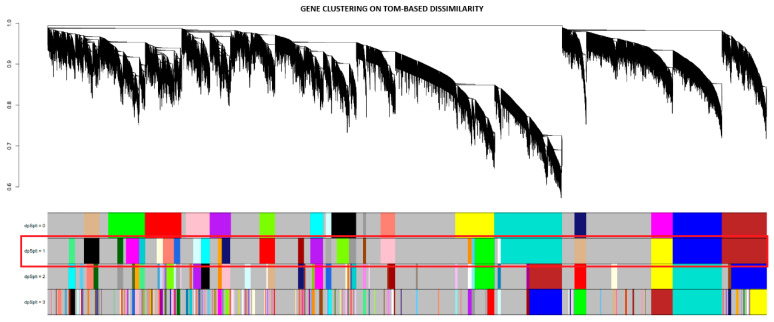
PD dendrogram of gene clustering using TOM-based dissimilarity and the visualization of the different module split sensitivities for hybrid tree cutting. The red box indicates the chosen deep split parameter, 1, and its resulting modules indicated by the different colors.

**Figure 4:**
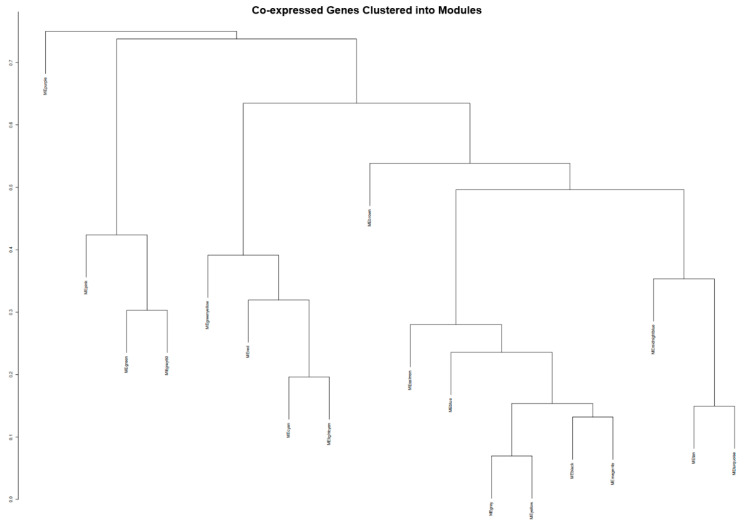
The dendrograms of the co-expressed genes clustered into their representative modules. Modules under the same branches have relatively similar co-expression patterns but are not as comparable to the co-expression patterns of genes within each module.

The modules in the PD gene clustering dendrogram were identified using the “hybrid tree cutting” function, which is part of the dynamic tree cutting family of algorithms but includes additional functionality to handle large datasets more efficiently [33]. Deep split arguments from 0 to 3 were used to control the sensitivity of the dynamic tree-cutting algorithm, as shown in Figure 3. Using a deep split argument of 1, to obtain a smaller number of larger modules, WGCNA was able to identify 18 gene co-expression modules designated by unique colors (Figure 4). The black module contained 911 genes; the blue module had a total of 1814 genes; the brown module had 1646 genes; cyan module contained 465 genes; the green module contained 1357 genes; the green-yellow module had 573 genes; the grey module, which contains genes that do not exhibit strong co-expression relationships in any other module, had a total of 2500 genes; grey60 contained 112 genes; the light-cyan module had 212 genes; the magenta module contained 786 genes; midnight-blue contained 429 genes; the pink module had 980 genes; the purple module had 786 genes; the red module had a total of 1350 genes; the salmon module contained 540 genes; the tan module had 570 genes; the turquoise module had a total of 2491 genes; and lastly, the yellow module contained 1452 genes.

#### 3.1.4. Module Preservation and Module Membership

**Figure 5:**
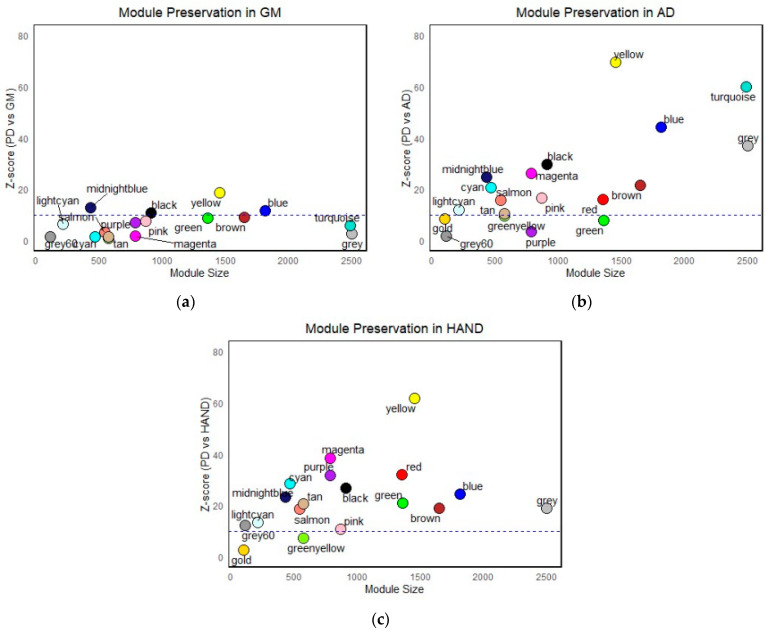
Module preservation analysis of the 18 identified gene co-expression modules from the PD network in the (**a**) glioblastoma dataset, (**b**) Alzheimer’s disease dataset, and (**c**) HIV-associated neurocognitive Disorders dataset. The dashed line at *Z* = 10 indicates the threshold for high module preservation. The PD modules midnight-blue, black, yellow, and blue all exhibit high preservation in GM, AD, and HAND.

The preservation of the identified modules in the PD gene co-expression network was quantified in GM, AD, and HAND using the Z-summary score, a measure of the density and connectivity of the modules [40]. As shown in Figure 5, module preservation varied greatly, with some being highly preserved across all datasets while others showed moderate and poor preservation. This suggests some aspects of the co-expression network were conserved across different diseases and others were unique, likely due to specific disease states and unique traits within each dataset. This study, however, focused only on the shared networks across the diseases, considering only the whole gene co-expression landscape without delving deeper into specific disease states or phenotypes of each disease.

Therefore, only modules with high preservation (z > 10) across all datasets, given by black, blue, midnight-blue, and yellow modules, were considered modules of interest and taken for further analysis. A summary of the z-score values for highly preserved modules across all datasets, including those that were only highly preserved in PD, AD, and HAND, is summarized in Table A1.

Moreover, module membership using in-module connectivity, kME (eigengene-based connectivity), was used to quantify each gene’s connectivity within a specific module. It is calculated as the correlation of the gene’s expression profile with the module eigengene, which is the first principal component of the module and can be considered a representative expression profile for each module [40,44]. The top genes within each module were ranked based on their kME values in each dataset. By considering genes with the highest maximum rank across all datasets, we ensure the presence of genes that are consistently highly connected within their respective modules across multiple datasets, suggesting key roles in biological mechanisms represented by these modules. A visual summary of the in-module connectivities of genes in the PD dataset against other diseases was presented using a scatterplot in Figure S1. All plots were presented with a positive correlation with *p*-values < 0.05 (Table S1).

### 3.2. Functional Annotation and Pathway Enrichment

**Figure 6:**
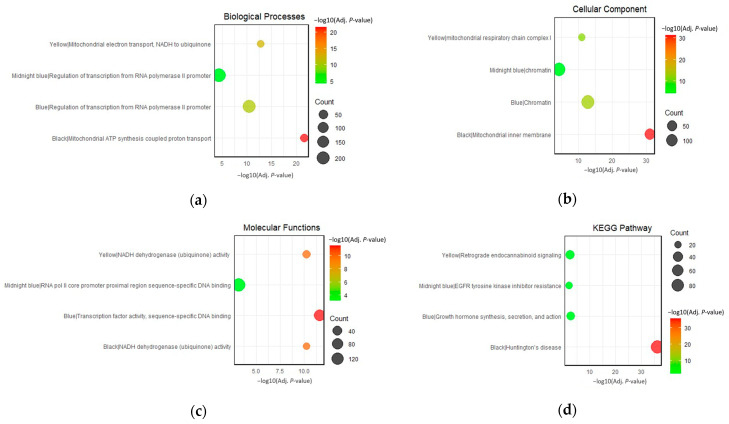
Top enriched terms for the black, blue, midnight-blue, and yellow modules in terms of (**a**) biological processes, (**b**) cellular components, (**c**) molecular functions, and (**d**) KEGG pathways.

To characterize the modules of interest, the top genes within each module were sent to the DAVID web server for functional annotation clustering. This allowed the grouping of terms that share biological attributes, such as involvement in the same biological processes, molecular functions, cellular components, and KEGG pathways, into clusters. The top-enriched GO terms for each module are presented in Figure 6a–c. KEGG pathways enriched only in the same cluster with top GO terms were prioritized (Figure 6d). Interestingly, several annotation clusters with significant enrichment scores that contain associations with neurodegeneration were identified. These clusters provide additional insights into the associations of the gene co-expression modules in the pathogenesis of HAND, AD, PD, and GM. The complete list of the significantly enriched clusters can be found in Supplementary Tables S2–S5.

### 3.3. Protein-Protein Interaction Networks and Identification of Hub Genes

**Figure 7:**
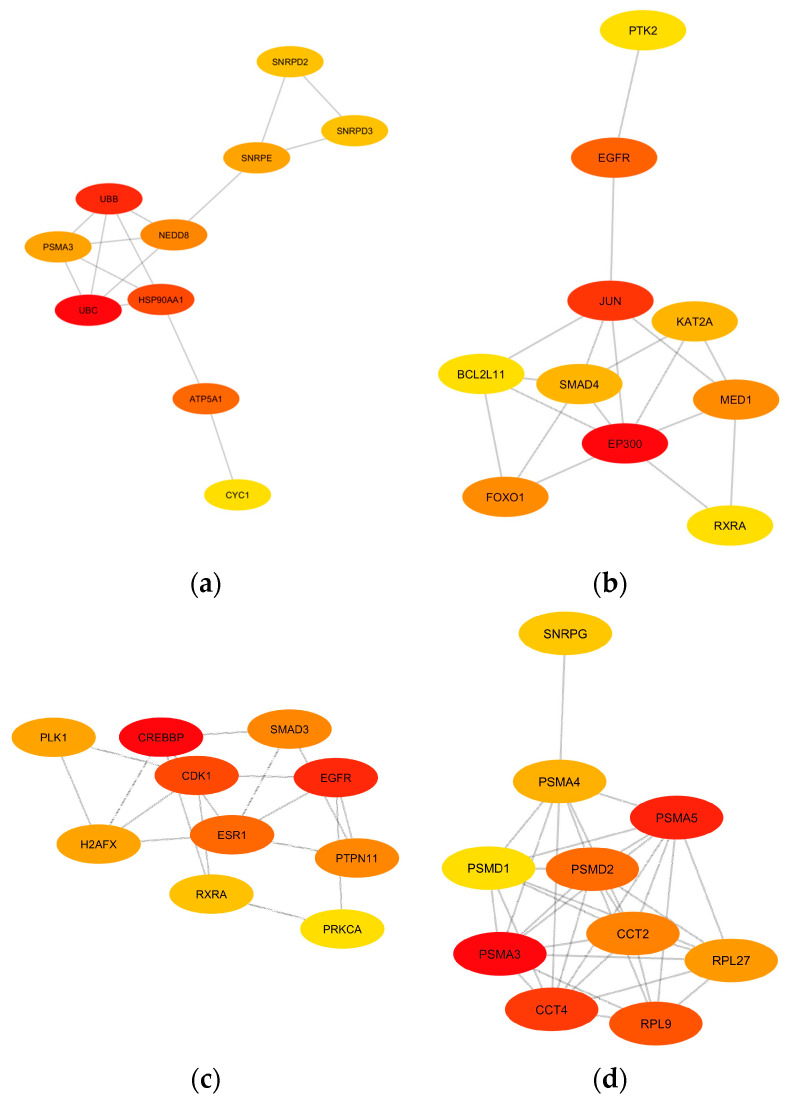
The identified top 10 hub gene networks based on the PPI networks of the (**a**) yellow module, (**b**) midnight-blue module, (**c**) blue module, and (**d**) black module are visualized based on degree. The color intensity of yellow to red indicates the rank of each node from lowest to highest, respectively.

To further explore the potential interactions among the genes and their corresponding proteins within the identified modules, PPI networks were constructed for each module of interest using the STRING database. A high confidence score (0.7) was set to ensure the reliability of the predicted interactions [37]. To pinpoint central genes in each PPI network, each network was imported to Cytoscape for the calculation of network and node scores, and CytoHubba was employed to rank nodes within a network using three (3) topological algorithms: degree, EPC, and MNC, thereby identifying potential hub genes. The 10 hub genes with the highest ranks for each network were extracted, providing a focused list of genes that potentially play pivotal roles in the network’s functionality and stability (Figure 7). The colors of each node correspond to the ranking of the hub genes, with the most intense red being the highest.

UBC, UBB, HSP90AA1, ATP5A1, NEDD8, PSMA3, SNRPE, SNRPD2, SNRPD3, and CYC1 were considered hub genes associated with the yellow module. The midnight-blue module contains the hub genes EP300, JUN, EGFR, MED1, FOXO1, KAT2A, SMAD4, RXRA, BCL2L11, and PTK2. The hub genes in the blue module are CREBBP, EGFR, CDK1, ESR1, SMAD3, PTPN11, H2AFX, PLK1, RXRA, and PRKCA. Lastly, the black module has PSMA3, PSMA5, PSMA4, CCT4, CCT2, RPL9, PMSD1, PMSD2, RPL27, and SNRPG as hub genes. The function of each individual hub gene is summarized in Table A7, Table A8, Table A9 and Table A10. These hub genes, given their central positions within the PPI network of each module of interest, warrant further investigation for their potential roles in the pathophysiology of the diseases under study.

### 3.4. Signature-Based Approach for Drug Repurposing

The top 10 hub genes of each module of interest were used as gene signatures and sent to the DRE web server for drug repurposing analysis. Drugs with negative Tau score values, down to −100, indicate the most significant associations with the submitted gene signature. The five (5) identified drug candidates with the lowest Tau scores were given emphasis, and their corresponding mechanisms of action are summarized in Table A6. The top-ranking drug candidate for modulating the upregulated hub genes was Dorzolamide, while Oxybuprocaine was the top-ranking drug candidate for the downregulated hub genes (Table 2). 

## 4. Discussion

### 4.1. Shared Gene Co-Expression Modules in PD, GM, HAND, and AD

The construction of gene co-expression networks to identify clusters of highly correlated genes is a powerful systems biology method that has been recently used in various biological contexts to identify disease-related genes, pathways, and networks [48]. One of the strengths of WGCNA is its ability to capture the complex interplay among genes, which is often missed by traditional differential expression analyses that consider each gene independently [49]. This makes WGCNA particularly suitable for the study of complex brain diseases that cause neurocognitive impairment, which is likely to involve the dysregulation of interconnected networks of genes rather than individual genes [21,50,51,52]. In the context of neurocognitive impairment, which refers to deficits in cognitive functions like language, memory, attention, and problem-solving due to underlying neurological abnormalities or dysfunction [53], Neurocognitive impairment is a hallmark of many brain disorders [53,54]. These conditions are characterized by intricate molecular and cellular dysregulations that manifest as cognitive decline. Traditional gene expression analyses may not fully capture the complexity of these dysregulations, as they often focus on individual genes rather than the networks they form [40].

WGCNA has gained popularity due to its robustness, the ability to capture soft thresholding for the approximation of scale-free networks, and the comprehensive framework it provides for network analysis, capturing the overall gene expression landscape [40]. The weighted gene co-expression modules black, blue, midnight-blue, and yellow were identified through the WGCNA and module preservation analysis of microarray disease datasets associated with neurocognitive impairments: HIV-associated neurocognitive disorder (GSE35864), Alzheimer’s disease (GSE5281), Parkinson’s disease (GSE7621), and glioma (GSE15824). These modules were identified to be highly preserved across all the disease datasets, suggesting strong associations with disease mechanisms shared across the four diseases. Particularly, the black and yellow modules are strongly associated with mitochondrial dysfunction and protein aggregation. The blue and midnight-blue modules display strong associations with transcriptional dysregulations related to neurodegenerative diseases. Moreover, it is crucial to differentiate between the pathomechanisms that underlie neurodegenerative disorders and gliomas. In the context of CNS tumors, including gliomas, protein misfolding often leads to aberrant signaling pathways that promote cell proliferation and survival, thereby contributing to tumorigenesis [12]. The balance between apoptosis and cell proliferation is indeed a key difference between these two disease categories. In neurodegenerative diseases, the emphasis is on the loss of neurons and the failure of cellular repair mechanisms, leading to a decline in cognitive and motor functions. In contrast, CNS tumors are characterized by uncontrolled cell growth and a failure of apoptosis, leading to mass formation and subsequent neurological symptoms [32]. It is worth noting that the intersection of these two seemingly disparate biological processes is not without precedent. Recent research has indicated that certain molecular pathways, such as the p53 and Wnt signaling pathways, are implicated in both neurodegeneration and CNS tumors [32,55].

### 4.2. Implications of Mitochondrial Dysfunction and Protein Aggregation on the Neurocognitive Impairment Network

Mitochondrial dysfunction is a key factor in the pathogenesis of various neurodegenerative diseases. Mitochondria are responsible for producing most of the cell’s energy through the process of oxidative phosphorylation, and their dysfunctionality has been studied to contribute to the progression of dementia in many neurodegenerative diseases such as Alzheimer’s, Parkinson’s, and Huntington’s disease [16,17]. Mitochondrial dysfunction can lead to increased production of reactive oxygen species (ROS), decreased ATP production, and the release of pro-apoptotic factors, all of which can contribute to neuronal cell death [14]. Impairment of the electron transport chain leads to cellular energy failure, which is especially detrimental to neurons as these cells have high energy demands [6]. Mitochondrial dysfunction is a prominent feature in the black and yellow modules, as evident in functional annotation clustering. The enrichment of terms in the top-scoring clusters “ubiquinone activity”, “mitochondrial ATP synthesis”, mitochondrial electron transport”, “mitochondrial inner membrane”, and “mitochondrial respiratory chain complex I” underscores the importance of mitochondrial health in neuronal function, playing a crucial role in maintaining synaptic activity and neuronal survival [56]. Moreover, impaired mitochondrial function can exacerbate protein misfolding, promote inflammation, and contribute to neuronal loss [6]. For instance, in Parkinson’s disease, mutations in *PINK1* and Parkin protein, which are crucial for maintaining mitochondrial health, are known to contribute to disease pathogenesis [6,15].

Other than top-scoring clusters, significantly enriched clusters within the black module also highlight the overrepresentation of terms associated with protein misfolding and aggregation. Aberrant protein folding and aggregation are hallmark features of neurocognitive impairment and neurodegeneration, contributing to cellular toxicity and neuronal death [57,58]. Proteins with complex structures, such as those involved in mRNA splicing, are particularly susceptible to misfolding. Once misfolded, these proteins can aggregate and form insoluble deposits, which can disrupt cellular function and trigger neuronal death [59]. The spliceosome, a dynamic complex of small nuclear ribonucleoproteins (snRNPs) and numerous proteins, orchestrates the precise removal of introns from pre-mRNA, a critical step in the maturation of mRNA and subsequent protein synthesis. The enrichment terms, such as mRNA splicing via the spliceosome and spliceosomal complex assembly, hint at a profound connection between the splicing machinery and the progression of neurodegeneration [60]. The meticulous assembly and function of the spliceosome involve a series of catalytic steps and the formation of complexes like the U2-type precatalytic spliceosome and the catalytic step 2 spliceosome. Any aberration in these processes can potentially lead to the generation of aberrant mRNA transcripts, which can translate into misfolded proteins, setting the stage for protein aggregation [61]. 

Several significantly enriched clusters of the yellow module also emphasize strong associations with protein misfolding and aggregation. The overrepresentation of the term proteasome core complex holds significant relevance. The proteasome is a crucial piece of cellular machinery responsible for the degradation of misfolded proteins and maintaining protein homeostasis within the cell. In the context of neurodegeneration, the impairment of the proteasomal system can lead to the accumulation of protein aggregates, a hallmark of several neurodegenerative conditions such as Alzheimer’s and Parkinson’s disease [58]. The intricate balance of protein synthesis and degradation, as indicated by terms like post-translational protein modification regulation of the cellular amino acid metabolic process, further underscores the critical role of protein homeostasis in preventing neurodegeneration [56,60,62]. The Wnt signaling pathway was also highlighted in the yellow module. Some components of the Wnt pathway, such as GSK-3β, are involved in both cancer and neurodegeneration but serve different roles. In cancer, GSK-3β is often inactivated to allow for Wnt pathway activation, while in Alzheimer’s disease, GSK-3β is overly active, leading to increased tau phosphorylation and aggregation [12,32,37]. 

Cell cycle regulation is another critical aspect emphasized in the yellow module. While neurons are typically considered post-mitotic, emerging evidence suggests that cell cycle re-entry can occur in neurodegenerative diseases, leading to neuronal death [63,64,65]. Lastly, the yellow module brings attention to the role of “retrograde endocannabinoid signaling,” a critical modulator of synaptic activity. Endocannabinoids are lipid signaling molecules that can travel in a retrograde manner from post-synaptic neurons to pre-synaptic neurons to bind to presynaptic cannabinoid receptors [66]. Binding inhibits the release of neurotransmitters, thereby modulating neuronal excitability and synaptic plasticity. Dysregulation of endocannabinoid signaling has been implicated in various neurological conditions. The enrichment of this pathway in the yellow module suggests a potential role in neurodegeneration, possibly through the modulation of synaptic activity and neuronal survival. In the context of neurodegeneration, alterations in endocannabinoid signaling could impact neuronal excitability, synaptic plasticity, and neuroinflammation, all of which are critical factors in disease progression [66].

### 4.3. Transcriptional Dysregulation in the Neurocognitive Impairment Network

The blue and midnight-blue modules, both enriched for the biological process “regulation of transcription from RNA polymerase II promoter," highlight the significance of transcriptional regulation in neurodegeneration. This is consistent with the growing body of literature that highlights the role of transcriptional dysregulation in the progression of neurocognitive impairment in brain diseases [50,67,68,69]. For instance, in Alzheimer’s disease, aberrant gene expression patterns have been linked to disease progression, with a notable shift in transcriptional priorities from synaptic function to immune response [51]. 

The enrichment of the cellular component “chromatin” in both modules further emphasizes the importance of chromatin remodeling in transcriptional regulation [70]. Disruptions in chromatin structure, which can lead to aberrant gene expression, have been implicated in neurodegenerative diseases [71]. This suggests that the integrity of chromatin structure and its role in facilitating or hindering transcription are crucial aspects of neuronal health and function. The blue module’s enrichment for the KEGG pathway, growth hormone synthesis, secretion, and action suggests a potential role of growth hormone-related processes in neurodegeneration. This aligns with studies showing that growth hormone therapy can improve cognitive function in growth hormone-deficient adults, indicating a potential neuroprotective role for growth hormone [72].

The midnight-blue module, while sharing similarities with the blue module, presents unique features. The enrichment of the molecular function, RNA polymerase II core promoter proximal region sequence-specific DNA binding, suggests a specific role in the initiation stages of transcription regulation. This could indicate a potential mechanism by which transcriptional dysregulation occurs in neurodegenerative diseases, possibly through alterations in the recruitment or activity of transcription factors in the core promoter region. Mutations or dysregulations in the genes encoding for transcription factors that bind to the core promoter proximal region can potentially lead to aberrant gene expression [73]. Recent studies have highlighted that mutations and aggregation of transcription factors such as TATA-binding protein (TBP), a component of the RNA polymerase II pre-initiation complex, are associated with Huntington’s disease [74,75]. These mutations can affect the binding affinity of TBP to the core promoter region, thereby altering the transcriptional landscape of neuronal cells. 

Furthermore, the enrichment of the KEGG pathway “Th1 and Th2 cell differentiation” in the midnight-blue module suggests the involvement of immune processes, specifically T cell differentiation. This aligns with the recognition of neuroinflammation in the black and yellow modules. The shift towards a pro-inflammatory state observed in diseases like Alzheimer’s, characterized by increased activation of microglia and astrocytes and increased production of pro-inflammatory cytokines, is thought to contribute to neuronal damage and disease progression.

These identified key molecular mechanisms shared across the brain disorders focused on in this study corroborate well with numerous studies surrounding neurocognitive impairment. The focus herein now shifts the outlook toward the proteins encoded by the key hub genes based on PPI networks in each module of interest.

### 4.4. Hub Genes in the Neurocognitive Impairment Network

Hub genes from PPI networks generated from robust WGCNA data can act as potential targets for repositioned drugs. In the yellow module, the ubiquitin proteins represented by *UBC* and *UBB* play a pivotal role in the ubiquitin-proteasome system (UPS), responsible for protein degradation within cells. Accumulation of ubiquitinated proteins is a hallmark of neurodegenerative pathologies, suggesting a failure in the proteasomal degradation pathway [76]. Moreover, mutations in *UBB* have been associated with the formation of neurofibrillary tangles in Alzheimer’s disease [77]. Given the accumulation of ubiquitinated proteins in these diseases, strategies to enhance UPS function or modulate ubiquitination processes are being explored as therapeutic avenues. For instance, small molecules that can modulate ubiquitin ligase activity are under investigation [55]. The heat shock protein 90 alpha family, class A member 1 (*HSP90AA1*), another highly ranked gene in the yellow module, acts as molecular chaperones, assisting in protein folding and preventing protein aggregation [78]. The overexpression of *HSP90AA1* has been observed in Alzheimer’s disease, and the use of inhibitors, geldanamycin and its analogs, has been proposed as a potential therapeutic strategy due to its role in tau stabilization and amyloid-beta aggregation, a hallmark feature of this disease [79]. 

The Proteasome 20S Alpha subunits, including *PSMA3*, *PSMA5*, and *PSMA4*, are the top hub genes in the black module. The proteasome complex, a part of the ubiquitin-proteasome system, is crucial for maintaining cellular homeostasis. For instance, *PSMA3* has been associated with altered proteasomal activity in Alzheimer’s disease. Given the central role of the ubiquitin-proteasome system in protein degradation, targeting its components has been a strategy in neurodegenerative diseases [80]. Bortezomib, a proteasome inhibitor, has been explored for its potential neuroprotective effects in models of Parkinson’s disease. While primarily used in oncology, the rationale for its use in neurodegenerative conditions stems from its ability to modulate proteasomal activity, which might aid in clearing protein aggregates [81]. Interconnections among these genes hint at a complex interplay between protein synthesis (ribosomal proteins), protein folding (chaperonins), and protein degradation (proteasome subunits and assembly proteins).

The top-ranking hub gene in the midnight-blue module is the E1A Binding Protein (*EP300*), which encodes the p300 protein and plays a pivotal role in transcriptional regulation through histone acetylation [82]. Dysregulated *EP300* activity has been linked to neurodegenerative diseases, particularly Alzheimer’s disease, where its hyperacetylation of tau, a microtubule-associated protein, has been observed, suggesting a potential mechanism for tauopathy, the aggregation of aberrant tau proteins in the brain [83]. Inhibitors have been explored as potential therapeutic agents; for example, a past study has shown that specific *EP300* inhibitors can mitigate tauopathy and associated cognitive deficits in cellular models of Alzheimer’s disease [84]. Additionally, elevated levels of the Jun Proto-Oncogene (*JUN*), the second-ranked hub gene in this module, have been observed in neurodegenerative conditions like Parkinson’s and Alzheimer’s disease. Primarily recognized as a proto-oncogene, *JUN* encodes for the c-Jun protein, a component of the *AP-1* transcription factor complex that plays a significant role in cell proliferation, differentiation, and apoptosis [85]. Its aberrant expression and mutations have been associated with the promotion of tumorigenic processes, contributing to the progression of glioblastoma [86]. In the context of neurocognitive impairment, the c-Jun protein is known to be involved in neuronal plasticity and regeneration, indicating a possible role in maintaining neuronal health [87]. Moreover, recent studies have suggested that the *JUN* gene might be implicated in the inflammatory responses observed in neurodegenerative conditions, potentially through its involvement in regulating cytokine production and immune responses [87]. 

The cAMP-response element binding protein (*CREBBP*), a transcriptional coactivator with histone acetyltransferase activity, is the top-ranking hub gene in the blue module. In Alzheimer’s disease, dysregulation of *CREBBP* has been linked to synaptic plasticity alterations [88]. Moreover, mutations in *CREBBP* have been associated with cognitive impairment, suggesting its pivotal role in maintaining neuronal health. Histone deacetylase inhibitors, which can indirectly modulate *CREBBP* activity, have been explored for their neuroprotective effects, as observed in models of Huntington’s disease [89]. Interestingly, due to the presence of glioma in the datasets studied herein, shared networks between neurodegeneration and cancer were also inferred. For instance, these hub genes are not only implicated in the pathogenesis of neurocognitive impairment but may also provide insights into other cancer diseases. The persistent activation of STAT3, a downstream target of *HSP90*, is known to promote cell proliferation and survival in cancer, making it a promising target for cancer therapy [90]. Similarly, *EP300* and *CREBBP* have been implicated in prostate cancer, with the former identified as an oncogene in a significant proportion of tumors.

More importantly, the findings herein show that the identified modules and hub genes are mostly well-associated with certain brain disorders like Alzheimer’s and Parkinson’s disease, at least to the extent of our literature search. It is worth noting that the constructed weighted gene co-expression modules and hub genes emphasize that these are shared across many brain disorders that lead to neurocognitive impairment, even with differing causes or pathophysiology, as evident in the inclusion of HIV-associated Neurocognitive Disorder and glioma datasets and their high module preservation scores.

### 4.5. Potential Drugs for Neurocognitive Impairment

Once the disease-associated gene co-expression modules are identified through WGCNA, they can be pipelined to drug-repurposing techniques, for instance, the virtual screening of compounds based on their molecular and gene signatures. In the context of utilizing gene signatures, the process begins with the identification of a specific set of genes (gene signatures) that are significantly altered in a particular disease state, in this case, the identified hub genes shared across HAND, AD, PDD, and glioma (Table A7, Table A8, Table A9 and Table A10). Once these gene signatures are identified, they can be used to search for known drugs that match the gene signatures of drugs in databases [50]. This is often achieved through computational approaches, where the gene signatures are compared with gene expression profiles induced by various drugs in large databases, such as the Connectivity Map (CMap) and the molecular signatures database (MsigDB) [91,92]. The underlying principle is that drugs modulating the disease-associated gene expression patterns might have therapeutic potential [27]. 

The top-ranking drug for the upregulated hub genes, Dorzolamide, is an inhibitor of carbonic anhydrase and is primarily used to treat glaucoma. Its main mechanism of action is the inhibition of carbonic anhydrase, an enzyme responsible for the reversible hydration of carbon dioxide [93]. By inhibiting this enzyme, Dorzolamide reduces the production of bicarbonate ions and protons, influencing intracellular pH. Dysregulated cellular pH has been implicated in Alzheimer’s disease. For instance, amyloid-beta peptides, which aggregate and form plaques in the brain, have been shown to disrupt cellular pH homeostasis, leading to neuronal dysfunction [94]. By modulating pH, Dorzolamide could potentially counteract this disruption, restoring neuronal function and reducing neurotoxicity. Although direct studies on its repurposing for neurodegeneration are limited, carbonic anhydrase inhibitors have shown potential for reducing oxidative stress, a key player in neurodegenerative processes. Beyond its primary mechanism, Dorzolamide has been shown to exert neuroprotective effects [95]. In models of retinal ganglion cell death, a process that shares similarities with neurodegenerative mechanisms, Dorzolamide demonstrated protective effects, potentially through the reduction of oxidative stress [96,97]. By reducing oxidative stress, Dorzolamide could mitigate neuronal damage and cell death. 

The top-ranking drug for modulating the downregulated hub genes is Oxybuprocaine, primarily used as a local anesthetic agent. It acts by blocking sodium channels, preventing the initiation and transmission of nerve impulses. Sodium channel dysregulation has been implicated in various neurodegenerative conditions, contributing to neuronal excitotoxicity. By modulating sodium channel activity, Oxybuprocaine might offer neuroprotective effects, reducing excitotoxic damage to neurons. Neuronal excitotoxicity, resulting from excessive glutamate release and subsequent overactivation of its receptors, is a well-established mechanism contributing to neuronal death in Alzheimer’s and Huntington’s disease [18,89]. Oxybuprocaine, as a sodium channel blocker, can potentially mitigate this excitotoxicity. Inhibiting the sodium channels can prevent excessive neuronal firing and the subsequent calcium influx that would otherwise lead to excitotoxic damage [98,99]. Moreover, microglial activation and the release of pro-inflammatory cytokines can exacerbate neuronal damage, leading to neuroinflammation. Some local anesthetics have demonstrated anti-inflammatory properties, and it is plausible that Oxybuprocaine might exert similar benefits, potentially contributing to its neuroprotective effects [100]. However, it is still crucial to approach these potential benefits with caution. While drug repurposing offers the advantage of utilizing agents with known safety profiles, the transition from one therapeutic context to another requires rigorous investigation. Further validation steps and in-depth clinical studies are essential to validate Dorzolamide’s and Oxybuprocaine’s efficacy and safety in the context of neurocognitive disorders. In future studies, further cross-study WGCNA can be conducted to construct gene co-expression networks that can discriminate between various traits or sample groups within each dataset. This allows the generation of modules and hub genes that depict a more detailed underlying biology of disorders and can be correlated to specific traits or conditions. 

## 5. Conclusions

This study identified highly preserved gene co-expression modules across Alzheimer’s disease (AD), Parkinson’s disease with Dementia (PDD), HIV-associated neurocognitive disorders (HAND), and glioma, diseases that cause neurocognitive impairment. This highlights the potential of the Weighted Gene Co-expression Network Analysis (WGCNA) approach in elucidating the complex molecular underpinnings of neurocognitive impairment, especially in the context of mitochondrial dysfunction and protein aggregation, which were supported by the characterization of correlated gene clusters and modules that were found to be highly preserved across the HAND, AD, PD, and glioma gene co-expression networks. By employing WGCNA and module preservation analysis to construct protein-protein interaction (PPI) networks, the findings in this study suggest that molecular mechanisms only evident in PDD and AD were shared across diseases like glioma and HAND, which hints that the identified disease mechanisms are hallmarks that could be shared across many disorders that lead to neurocognitive impairment, as substantially corroborated in both past and recent findings. 

More importantly, critical hub genes that play central roles across these shared modules were determined, suggesting their potential as therapeutic targets. These hub genes underscore their possible intricate balance of cellular processes for neurocognitive function, and their dysregulation may have profound implications for disease mechanisms that cause its impairment, given their critical roles in protein homeostasis, energy metabolism, cell signaling, and gene expression. This approach offers insights into the exploration of potential repurposable drugs that are significantly associated with the hub genes shared across different brain disorders. Although the availability of comparable gene expression datasets for brain diseases is a critical factor in gene co-expression analysis studies, setting a direction for meta-analytical WGCNA studies contributes to improving our understanding of the shared gene co-expression landscape in neurocognitive impairment and paving the way for the development of more effective and targeted drugs. 

## Figures and Tables

**Table 1:** Summary of GEO Datasets.

	HAND (GSE35864)	AD (GSE5281)	PD (GSE7621)	GM (GSE15824)
Conditions	Control (18), HIV *w*/*o* NCI (18), HIV w/dementia *w*/*o* HIVE (21), HIV w/NCI and HIVE (15)	Control (74), Alzheimer’s disease (87)	Control (9), Parkinson’s disease (16)	GBM (12), GBM-2 (3), Astro (8), and Oligo (7)
Type	Expression profiling by array			
Platform	GPL570—HG-U133 Plus 2 Affymetrix Human Genome U133 Plus 2.0 Array			
Sample Type	Post-mortem brain tissue			Frozen gliomas tissue samples
RNA Source	basal ganglia, white matter, and frontal cortex	EC, HC, PC, VC, medial temporal gyrus, and superior frontal gyrus	Substantia nigra (basal ganglia)	Glioma tissue
No. of Samples	72	161	25	35

Human Immunodeficiency Virus (HIV), HIV-associated Neurodegenerative Disorder (HAND), Neurocognitive Impairment (NCI), HIV-associated Encephalitis (HIVE), Alzheimer’s Disease (AD), Parkinson’s Disease (PD), Glioma (GM), Primary Glioblastoma (GBM), Secondary Glioblastoma (GBM-2), Astrocytoma (Astro), Oligodendroglioma (Oligo), Entorhinal Cortex (EC), Hippocampus (HC), Posterior Cingulate (PC), and Primary Visual Cortex (VC).

**Table 2:** Top drug candidates for upregulated and downregulated hub genes.

Expression	Genes	Drug	Known Mechanism	Tau	FDR
Upregulated	*PSMA3*, *PSMA5*, *CCT4*, *CCT2*, *PSMD1*, *CDK1*, *ESR1*, *SMAD3*, *PTK2*, *UBC*, *HSP90AA1*, *ATP5A1*, *NEDD8*, *PSMA3*, *SNRPE*, *SNRPD2*, *SNRPD3*, *CYC1*	Dorzolamide	Carbonic anhydrase inhibitor	−99.75	2.78 × 10^−3^
Downregulated	*CREBBP*, *EGFR*, *PTPN11*, *H2AFX*, *RXRA*, *EP300*, *JUN*, *EGFR*, *MED1*, *FOXO1*, *SMAD4*, *RXRA*	Oxybuprocaine	Local anesthetic	−99.76	3.64 × 10^−3^

**Figure A1:**
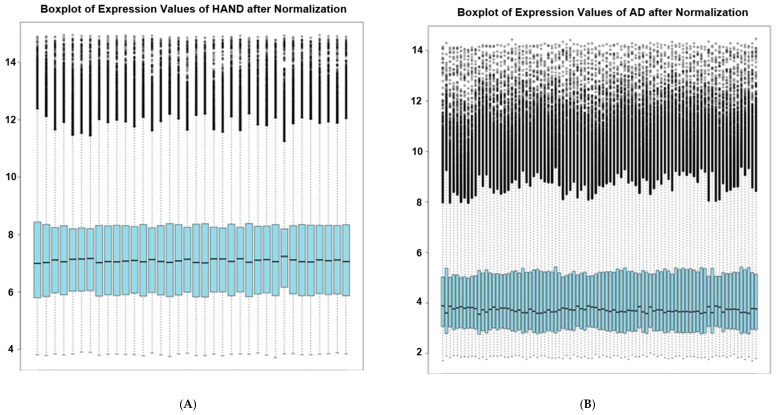

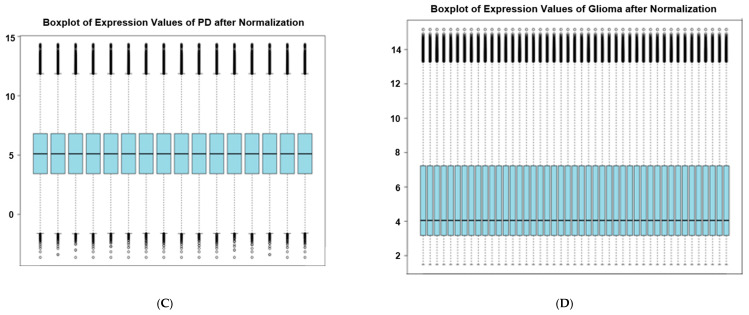
Boxplot of microarray datasets (**A**) HAND dataset, (**B**) AD dataset, (**C**) PD dataset, and (**D**) Glioma dataset after normalization and filtering steps. RMA normalization was performed, control samples were omitted, and genes with missing symbols and gene expression values were removed.

## Data Availability

The microarray expression data used in this study are openly available in the Gene Expression Omnibus (GEO) database of the National Center for Biotechnology Information (NCBI) (https://www.ncbi.nlm.nih.gov/geo/ (accessed on 23 April 2023)) with accession IDs GSSE66354, GSE68848, GSE74195, and GSE43290.

## Supplementary Materials

The following supporting information can be downloaded: https://www.mdpi.com/article/10.3390/brainsci13111564/s1, Figure S1: Visual summary of in-module connectivities of genes in PD against HAND, AD, and GM; Table S1: Statistical summary of in-module connectivities of genes in PD against HAND, AD, and GM; Table S2: Top annotation clusters of the yellow module; Table S3: Top annotation clusters of the midnight-blue module; Table S4: Top annotation clusters of the blue module; Table S5: Top annotation clusters of the black module.

## Appendix A

**Table A1:** Highly preserved modules (Z > 10 in all datasets) and their corresponding top-ranking hub genes based on intramodular connectivity (kME).

	Module No.	Module	Size	Preservation (Z-Score)	Top Hub Gene
Highly preserved modules in all datasets	1	Black	911	10.97084	CENPBD1P
	2	Blue	1814	11.78024	PRR22
	12	Midnight blue	429	12.77744	DDR1
	19	Yellow	1452	18.77502	MSANTD3
Highly preserved modules in HIV-associated Neurocognitive Disorder, Alzheimer’s Disease, and Parkinson’s Disease datasets	3	Brown	1646	19.02149	DDR1
	4	Cyan	465	28.54539	AFG3L1P
	10	Light cyan	212	13.505348	CENPBD1P
	11	Magenta	786	38.505348	PRR22
	13	Pink	870	10.799127	DDR1
	15	Red	1350	32.136386	SCARB1
	16	Salmon	540	18.517416	MSANTD3
	17	Tan	570	20.749038	DDR1
	18	Turquoise	2491	84.408500	CORO6

**Table A2:** GO enrichment of highly preserved modules across all datasets in terms of biological processes. GO terms and their corresponding processes were obtained from the Gene Ontology Consortium (http://www.geneontology.org (accessed 23 August 2023)) [101].

Module	Top Enriched Biological Processes	Count	Adj. *p*-Value
Black	GO:0042776 mitochondrial ATP synthesis-coupled proton transport	28	2.24 × 10^−22^
Blue	GO:0006357 regulation of transcription from the RNA polymerase II promoter	183	3.03 × 10^−11^
Midnight blue	GO:0006357 regulation of transcription from the RNA polymerase II promoter	214	4.99 × 10^−05^
Yellow	GO:0006120 mitochondrial electron transport, NADH to ubiquinone	24	1.66 × 10^−13^

**Table A3:** GO enrichment is based on highly preserved modules across all datasets in terms of cellular components. GO terms and their corresponding components were obtained from the Gene Ontology Consortium (http://www.geneontology.org (accessed 23 August 2023)) [101].

Module	Top Enriched Cellular Components	Size	Adj. *p*-Value
Black	GO:0005743 mitochondrial inner membrane	73	6.37 × 10^−32^
Blue	GO:0000785 chromatin	149	1.88 × 10^−13^
Midnight blue	GO:0000785 chromatin	137	4.94 × 10^−05^
Yellow	GO:0005747 mitochondrial respiratory chain complex I	22	1.11 × 10^−11^

**Table A4:** GO enrichment is based on highly preserved modules across all datasets in terms of molecular function. GO terms and their corresponding functions were obtained from the Gene Ontology Consortium (http://www.geneontology.org (accessed 23 August 2023)) [101].

Module	Top Enriched Molecular Functions	Size	Adj. *p*-Value
Black	GO:0008137 NADH dehydrogenase (ubiquinone) activity	17	6.32 × 10^−11^
Blue	GO:0003700 transcription factor activity, sequence-specific DNA binding	99	2.33 × 10^−12^
Midnight blue	GO:0000978 RNA pol-II core promoter sequence-specific DNA binding	157	6.92 × 10^−04^
Yellow	GO:0008137~NADH dehydrogenase (ubiquinone) activity	21	6.32 × 10^−11^

**Table A5:** Pathway analysis on highly preserved modules across all datasets based on the KEGG pathway database. KEGG pathways were obtained from the Kyoto Encyclopedia of Genes and Genomes (KEGG) database (https://www.genome.jp/kegg (accessed 23 August 2023)) [102].

Module	Top Enriched KEGG Pathways	Size	Adj. *p*-Value
Black	hsa05016: Huntington’s disease	92	9.34 × 10^−37^
Blue	hsa04935: Growth hormone synthesis, secretion, and action	25	1.80 × 10^−03^
Midnight blue	hsa01521: EGFR tyrosine kinase inhibitor resistance	20	8.60 × 10^−03^
Yellow	hsa04723: Retrograde endocannabinoid signaling	27	3.78 × 10^−03^

**Table A6:** Summary of top drug candidates for modulating hub gene perturbations.

Expression	Genes	Drug	Mechanism	Tau	FDR
Upregulated	PSMA3, PSMA5, CCT4, CCT2, PSMD1, CDK1, ESR1, SMAD3, PTK2, UBC, HSP90AA1, ATP5A1, NEDD8, PSMA3, SNRPE, SNRPD2, SNRPD3, CYC1	Dorzolamide	Carbonic anhydrase inhibitor	−99.75	2.78 × 10^−03^
		Deracoxib	Cyclooxygenase inhibitor	−99.59	5.28 × 10^−06^
		Lofexidine	Adrenergic receptor agonist	−99.38	4.30 × 10^−03^
		Ranolazine	Sodium channel blocker	−99.22	2.01 × 10^−03^
		Methysergide	Serotonin receptor antagonist	−99.18	3.14 × 10^−03^
Downregulated	CREBBP, EGFR, PTPN11, H2AFX, RXRA, EP300, JUN, EGFR, MED1, FOXO1, SMAD4, RXRA	Oxybuprocaine	Local anesthetic	−99.76	3.64 × 10^−03^
		Methocarbamol	Muscle relaxant	−99.46	6.44 × 10^−03^
		Hydrocortisone	Glucocorticoid receptor agonist	−99.22	2.67 × 10^−04^
		Pentolinium	Cholinergic receptor antagonist	−98.91	9.46 × 10^−03^
		Ornidazole	Antiprotozoal agent	−98.86	9.92 × 10^−04^

**Table A7:** The top 10 hub genes in the yellow module were identified through PPI networks and their functions obtained from the GeneCards database (www.genecards.org (accessed 23 August 2023)) [103].

Gene	Function
UBC, UBB	Protein modification through covalent attachment to target proteins
HSP90AA1	Molecular chaperone for protein maturation/regulation
ATP5A1	Component of the mitochondrial ATP synthase Complex V
NEDD8	Cell cycle control, enhances ubiquitin ligase activity
PSMA3	Proteolytic degradation of intracellular proteins, protein homeostasis
SNRPE, SNRPD2, SNRPD3	Splicing of cellular pre-mRNAs, involved in the assembly of the heptameric protein ring on the small nuclear RNA
CYC1	Transfers electrons from the Rieske protein to Cytc, aiding in energy production.

**Table A8:** The top 10 hub genes in the midnight-blue module were identified through PPI networks and their functions obtained from the GeneCards database (www.genecards.org (accessed 23 August 2023)) [103].

Symbol	Function
EP300	Transcription regulation, non-histone protein acetylation
JUN	Transcriptional activation in certain cancer pathways
EGFR	G protein-coupled receptor signaling is involved in learning and memory functions
MED1	Transcription of RNA polymerase II-dependent genes
FOXO1	Metabolic homeostasis, oxidative stress response, redox balance, DNA repair in beta-cells, autophagic cell death
KAT2A	Promotes transcriptional activation and plays a role in chromatin remodeling
SMAD4	TGF-beta signal transduction, transcription regulation, tumor suppression,
RXRA	Regulates gene expression by binding to retinoic acid response elements
BCL2L11	Cell death regulation
PTK2	Cell migration/adhesion, cytoskeleton reorganization, nervous system development, apoptosis, signal transduction, and P53/TP53 activity regulation

**Table A9:** The top 10 hub genes in the blue module were identified through PPI networks and their functions obtained from the GeneCards database (www.genecards.org (accessed 23 August 2023)) [103].

Symbol	Function
CREBBP	Transcription, circadian transcriptional activation, nucleotide excision repair
EGFR	Activates RAS-RAF-MEK-ERK and PI3 kinase-AKT, influencing cellular responses and being involved in GPCR signaling for learning and memory functions
CDK1	Genome replication and cell death in postmitotic neurons
ESR1	Transcription regulations
SMAD3	Mediator in signal transduction, regulate transcription, tumor suppression
PTPN11	Signal transduction, MAPK signaling pathway, protein dephosphorylation
H2AFX	Cell cycle arrest, DNA double-strand break repairs
PLK1	Regulates centrosome maturation, spindle assembly, cohesin removal from chromosomes, mitotic exit, transcriptional regulation, and apoptosis
RXRA	Gene expression regulation by binding to retinoic acid response elements
PRKCA	Cell cycle progression, modulating cell motility, and tumor progression

**Table A10:** The top 10 hub genes in the black module were identified through PPI networks and their functions obtained from the GeneCards database (www.genecards.org (accessed 23 August 2023)) [103].

Symbol	Function
PSMA3, PSMA5, PSMA4	Proteolytic degradation of intracellular proteins and protein homeostasis mediate both ubiquitin-dependent and independent protein degradation
CCT4, CCT2	Molecular chaperones regulate protein folding and transport vesicles to the cilia.
RPL9	Large ribosomal subunit protein ul6; L ribosomal proteins
PMSD1, PMSD2	Protein degradation and homeostasis, cell progression/apoptosis, and DNA repair.
RPL27	Proper rRNA processing and maturation of 28S and 5.8S rRNAs.
SNRPG	Pre-mRNA splicing, assembly of a heptameric protein ring on small nuclear RNA
