# Supplementary material for: Navigating the Gene Co-Expression Network and Drug Repurposing Opportunities for Brain Disorders Associated with Neurocognitive Impairment
Source: Brain Sci. 2023 Nov 7;13(11):1564. doi: 10.3390/brainsci13111564 (PMC10669457; doi:10.3390/brainsci13111564)
Supplement: Supplementary file 1 [file brainsci-13-01564-s001.zip › brainsci-2642635-supplementary.pdf]

Supplementary Information

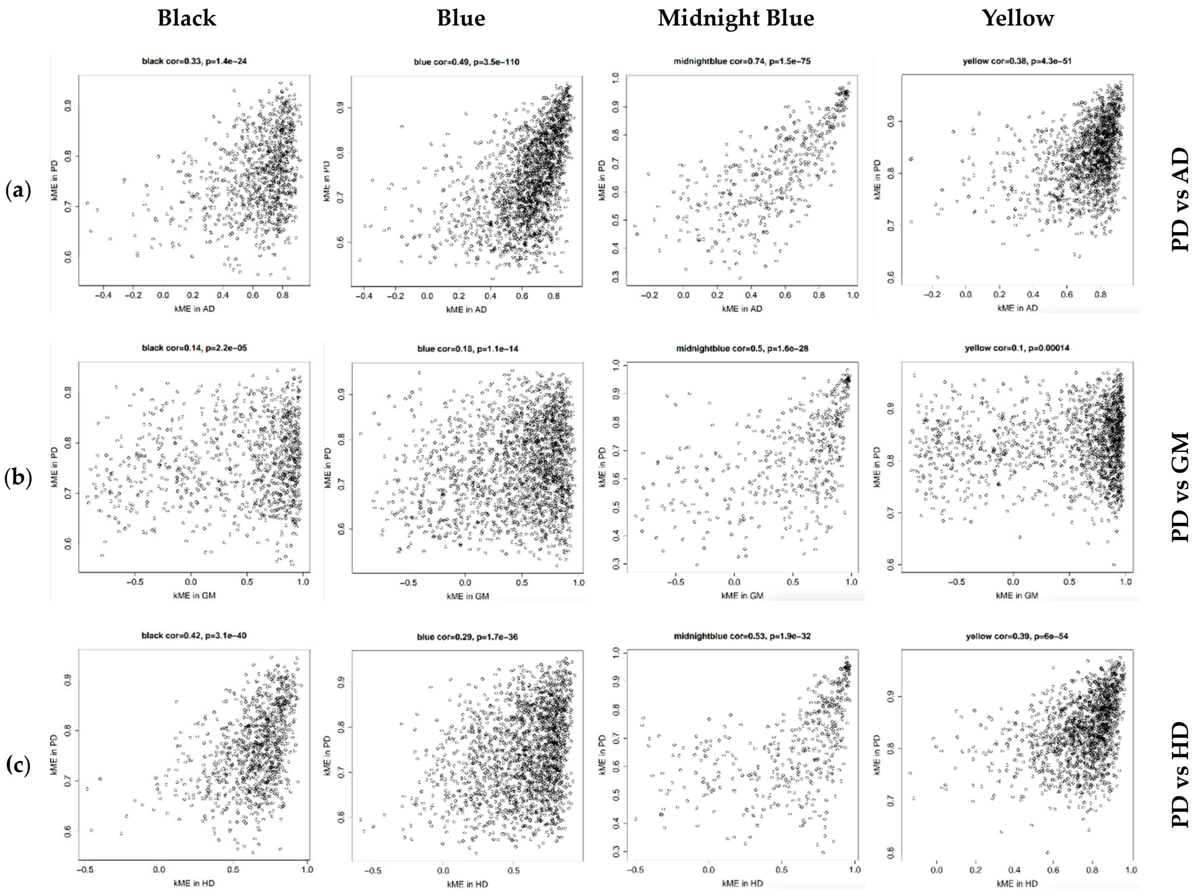

Figure S1. Visual summary of in-module connectivities, kME values, of genes in highly preserved modules across all datasets, in PD plotted against (**a**) AD, (**b**) GM, and (**c**) HD. Genes in the upper right section of the plot are likely to be hub genes.

Table S1. Statistical summary for in-module connectivities (kME) of genes of PD with GM, HD, AD datasets.

Module	PD vs GM		PD vs HD		PD vs AD	
	Correlation	P-value	Correlation	P-value	Correlation	P-value
**Black**	0.14	2.2E-05	0.42	3.1E-40	0.33	1.4E-24
**Blue**	0.18	1.1E-14	0.29	1.7E-36	0.49	3.5E-110
**Midnight Blue**	0.5	1.6E-28	0.53	1.9E-32	0.74	1.5E-75
**Yellow**	0.1	1.4E-4	0.39	6E-54	0.38	4.3E-51

Table S2. Top annotation clusters of the yellow module.

Cluster	Category	Term
1	KEGG	hsa05012: Parkinson's disease
	KEGG	hsa05020: Prion disease
	KEGG	hsa05022:Pathways of neurodegeneration - multiple diseases
	KEGG	hsa05010:Alzheimer disease
2	BP	GO:0006120 mitochondrial electron transport, NADH to ubiquinone
	CC	GO:0005747 mitochondrial respiratory chain complex I
	MF	GO:0008137 NADH dehydrogenase (ubiquinone) activity
	KEGG	hsa04723:Retrograde endocannabinoid signaling
3	CC	GO:0005839 proteasome core complex
	BP	GO:0036388 pre-replicative complex assembly
	BP	GO:0031145 anaphase-promoting complex-dependent catabolic process
	BP	GO:0006521 regulation of cellular amino acid metabolic process
	BP	GO:0002479 antigen processing and presentation of exogenous peptide antigen via MHC class I, TAP-dependent
	BP	GO:0061418 regulation of transcription from RNA polymerase II promoter in response to hypoxia
	BP	GO:1902036 regulation of hematopoietic stem cell differentiation
	BP	GO:0038095 Fc-epsilon receptor signaling pathway
	BP	GO:0010972 negative regulation of G2/M transition of mitotic cell cycle
	BP	GO:0070498 interleukin-1-mediated signaling pathway
	BP	GO:0002223 stimulatory C-type lectin receptor signaling pathway
	BP	GO:0060071 Wnt signaling pathway, planar cell polarity pathway
	BP	GO:0043687 post-translational protein modification
	BP	GO:0038061 NIK/NF-kappaB signaling
	BP	GO:0033209 tumor necrosis factor-mediated signaling pathway
	BP	GO:0090090 negative regulation of canonical Wnt signaling pathway
	BP	GO:0055085 transmembrane transport

Table S3. Top annotation clusters of the midnight-blue module.

Cluster	Category	Term
1	BP	GO:0006357 regulation of transcription from RNA polymerase II promoter
	CC	GO:0000785 chromatin
	MF	GO:0000978 RNA polymerase II core promoter proximal region sequence-specific DNA binding
	MF	GO:0003700 transcription factor activity, sequence-specific DNA binding
	MF	GO:0000981 RNA polymerase II transcription factor activity, sequence-specific DNA binding
	MF	GO:1990837 sequence-specific double-stranded DNA binding
2	BP	GO:0006468 protein phosphorylation
	MF	GO:0004712 protein serine/threonine/tyrosine kinase activity
	MF	GO:0004674 protein serine/threonine kinase activity
	MF	GO:0004672 protein kinase activity
3	KEGG	hsa04658:Th1 and Th2 cell differentiation
	KEGG	hsa04659:Th17 cell differentiation
	KEGG	hsa05235:PD-L1 expression and PD-1 checkpoint pathway in cancer

Table S4. Top annotation clusters of the blue module.

Cluster	Category	Term
1	CC	GO:0003700 transcription factor activity, sequence-specific DNA binding
	MF	GO:0045944 positive regulation of transcription from RNA polymerase II promoter
	BP	GO:1990837 sequence-specific double-stranded DNA binding
	MF	GO:0001228 transcriptional activator activity, RNA pol II transcription regulatory region sequence-specific binding
	MF	GO:0006357 regulation of transcription from RNA polymerase II promoter
	BP	GO:0000981 RNA polymerase II transcription factor activity, sequence-specific DNA binding
	MF	GO:0000978 RNA polymerase II core promoter proximal region sequence-specific DNA binding
	MF	GO:0003700 transcription factor activity, sequence-specific DNA binding
2	BP	GO:0006468 protein phosphorylation
	BP	GO:0018105 peptidyl-serine phosphorylation
	MF	GO:0004712 protein serine/threonine/tyrosine kinase activity
	MF	GO:0004674 protein serine/threonine kinase activity
	MF	GO:0004672 protein kinase activity
	MF	GO:0005524 ATP binding
3	KEGG	hsa04935:Growth hormone synthesis, secretion, and action
	KEGG	hsa04915:Estrogen signaling pathway
	KEGG	hsa04926:Relaxin signaling pathway
	KEGG	hsa05163:Human cytomegalovirus infection
	KEGG	hsa01522:Endocrine resistance

Table S5. Top annotation clusters of the black module.

Cluster	Category	Term
1	KEGG	hsa05016:Huntington disease
	KEGG	hsa05012:Parkinson disease
	KEGG	hsa05014:Amyotrophic lateral sclerosis
	KEGG	hsa05020:Prion disease
	KEGG	hsa00190:Oxidative phosphorylation
	KEGG	hsa05022:Pathways of neurodegeneration - multiple diseases
	CC	GO:0005743 mitochondrial inner membrane
	KEGG	hsa05010:Alzheimer disease
	BP	GO:0042776 mitochondrial ATP synthesis coupled proton transport
	KEGG	hsa05415:Diabetic cardiomyopathy
	KEGG	hsa05208:Chemical carcinogenesis - reactive oxygen species
	KEGG	hsa04714:Thermogenesis
	BP	GO:0009060 aerobic respiration
	KEGG	hsa04932:Non-alcoholic fatty liver disease
	BP	GO:0006120 mitochondrial electron transport, NADH to ubiquinone
	CC	GO:0005747 mitochondrial respiratory chain complex I
	MF	GO:0008137 NADH dehydrogenase (ubiquinone) activity
	BP	GO:0032981 mitochondrial respiratory chain complex I assembly
	KEGG	hsa04723:Retrograde endocannabinoid signaling
2	CC	GO:1904813 ficolin-1-rich granule lumen
	CC	GO:0034774 secretory granule lumen
	CC	GO:0005576 extracellular region
3	BP	GO:0000398 mRNA splicing, via spliceosome
	CC	GO:0005681 spliceosomal complex
	CC	GO:0071005 U2-type precatalytic spliceosome
	CC	GO:0071013 catalytic step 2 spliceosome
	KEGG	hsa03040:Spliceosome
	BP	GO:1903241 U2-type pre-spliceosome assembly
	CC	GO:0046540 U4/U6 x U5 tri-snRNP complex
	BP	GO:0008380 RNA splicing
	CC	GO:0005686 U2 snRNP
	CC	GO:0071007 U2-type catalytic step 2 spliceosome
	CC	GO:0071011 precatalytic spliceosome
	CC	GO:0034709 methylosome
	CC	GO:0005689 U12-type spliceosomal complex
	BP	GO:0000387 spliceosomal snRNP assembly
	CC	GO:0097526 spliceosomal tri-snRNP complex
	CC	GO:0005682 U5 snRNP
	CC	GO:0005687 U4 snRNP
	BP	GO:0036261 7-methylguanosine cap hypermethylation
	BP	GO:0000245 spliceosomal complex assembly
	CC	GO:0030532 small nuclear ribonucleoprotein complex
	CC	GO:0034715 pICln-Sm protein complex
